# Waggle Dance Distances as Integrative Indicators of Seasonal Foraging Challenges

**DOI:** 10.1371/journal.pone.0093495

**Published:** 2014-04-02

**Authors:** Margaret J. Couvillon, Roger Schürch, Francis L. W. Ratnieks

**Affiliations:** 1 Laboratory of Apiculture and Social Insects, School of Life Sciences, University of Sussex, Falmer, Brighton, United Kingdom; 2 Laboratory of Social Evolution, School of Life Sciences, University of Sussex, Falmer, Brighton, United Kingdom; Ghent University, Belgium

## Abstract

Even as demand for their services increases, honey bees (*Apis mellifera*) and other pollinating insects continue to decline in Europe and North America. Honey bees face many challenges, including an issue generally affecting wildlife: landscape changes have reduced flower-rich areas. One way to help is therefore to supplement with flowers, but when would this be most beneficial? We use the waggle dance, a unique behaviour in which a successful forager communicates to nestmates the location of visited flowers, to make a 2-year survey of food availability. We “eavesdropped” on 5097 dances to track seasonal changes in foraging, as indicated by the distance to which the bees as economic foragers will recruit, over a representative rural-urban landscape. In year 3, we determined nectar sugar concentration. We found that mean foraging distance/area significantly increase from springs (493 m, 0.8 km^2^) to summers (2156 m, 15.2 km^2^), even though nectar is not better quality, before decreasing in autumns (1275 m, 5.1 km^2^). As bees will not forage at long distances unnecessarily, this suggests summer is the most challenging season, with bees utilizing an area 22 and 6 times greater than spring or autumn. Our study demonstrates that dancing bees as indicators can provide information relevant to helping them, and, in particular, can show the months when additional forage would be most valuable.

## Introduction

Pollinating insects, including honey bees (*Apis mellifera*), continue to decline in Europe and North America [1]–[5], even though the demand for their services is increasing [6]–[8]. The number of managed hives in Great Britain has decreased 75% in the past century; in the United States, the 62% decline from 6 million in the 1940s to 2.3 million in 2008 is even more rapid [5], [9]. Honey bees face many challenges including pests [10], pathogens [11] and pesticides [12]. However, independent of these is another major issue affecting wildlife in general: landscape changes in the last century such as agricultural intensification have reduced flowers and flower-rich habitats that provide nectar and pollen for honey bees and other insects [13]–[18]. These changes are predicted to continue [19]. One suggestion on how to help bees is to provide more flowers when they are lacking [4], [9]. Although simple in principle, this is less easy in practice: when do bees most need additional flowers? To obtain directly data on the amount of forage available in a landscape-wide area, one could, with great effort, count competing flower-visiting insects and flowers and determine nectar and pollen availability. Perhaps this difficulty may explain why lack of forage is an often-mentioned reason behind bee declines [4], [9], but is relatively under-studied (although see Carvell et al. 2006 for bumble bees [15]).

The honey bee possesses a unique and fascinating behaviour in which a successful forager, upon returning to the hive, communicates to unemployed nestmate foragers the location of where she has collected food [20], [21]. The vector information is therefore available for eavesdropping researchers as a tool for ecology. Honey bees, as economically savvy foragers, weigh the relevant costs and benefits for that forage in their decision to recruit to the location [22]–[24], which makes the dance an integrative message that evaluates landscape profitability. Because honey bees are adept at scouting the landscape for food [25] and because flight is costly [23], foragers will not collect at long distances unnecessarily [20], [24]. Communicated distance therefore is a simple and powerful proxy for forage availability.

Previous work investigating recruitment distances using the waggle dance has focused on a few weeks or months of the much longer foraging year [26]–[29], most likely because dance decoding is time-costly and must be done by hand. However, an increased understanding of intra-dance variation has greatly streamlined the process [30], making it easier to decode high numbers of dances. Decoded dances provide a unique data set that is integrated and not confounded by weather and competition from other insects (see Discussion).

Here, for the first time, we investigate month by month and season by season variation in honey bee foraging distance over a representative rural-urban landscape. In year 3, we determined the nectar sugar concentration, which correlates with quality, returned by foragers. We found significant and consistent variation in mean foraging distance and area, where summer is the season in which bees must roam further and utilize a foraging area 22 and 6 times greater than spring or autumn respectively, even though they do not necessarily bring back better quality forage. More generally, our study demonstrates that honey bees may be used as indicators and can show through their dance the seasons in which forage is relatively less available and, by extension, when additional forage would be most beneficial.

## Materials and Methods

### Decoding honey bee waggle dances

The methods followed Couvillon et al. (2012) [30]. The honey bees used were of mixed European subspecies, but predominantly the British black bee *Apis mellifera mellifera*, and colonies were unrelated. The three colonies were held in observation hives of approximately 5000 workers at the University of Sussex, which is in the countryside 1 km NW of Brighton, a large city. The bees were allowed to forage naturally, and the potential foraging range [20], [27] contained a wide diversity of land types. Within a 4 km radius of the hives, these included agricultural land (62%, including both arable, improved grassland), urban and suburban areas (21%, including gardens, allotments, and built-up areas), broadleaved woodlands (10%), and unimproved grassland (7%). Similar land-use mosaics are widespread throughout the United Kingdom, North America, and most of western Europe [31].

All three colonies were queen-right and maintained throughout the duration of the project for swarm prevention and to keep the number of workers and amount of brood consistent. Although 5000 workers is smaller than what is found in a more traditional hive, it was shown that colony size (ranging from 6000–20,000 workers) does not significantly impact foraging distance [32]. Observation hives sometimes require food supplementation with sugar solution during periods of nectar dearth (e.g., July or in early spring when bad weather precludes foragers from collecting food for several consecutive days). On these occasions, we fed the colonies on Friday and did not resume data collection until the following Monday, which is more than sufficient time for the honey bees to drain the supplemental stores and to resume normal foraging (MJC personal communication). We only supplemented the hives with protein cake in February 2010 and 2011, before dances were recorded.

We video recorded dances within an area (25 cm×25 cm) for one hour on most days when the bees were foraging and then uploaded videos to iMac computers to decode the dances by playing the video frame by frame in Final Cut Express (version 4.0.1). We decoded four mid-dance, consecutive waggle runs [30], which repeat the same vector (direction+distance) multiple times within a dance. Waggle run duration in seconds (resolution: 1/25 second) was determined using the timer in the software. Angle in degrees was obtained using a protractor (maximum measurement error approximately 1°) against a vertical reference, created by plumb lines of fishing string with a washer at the end that were attached to each hive and visible as white lines on the video. We would note if the forager possessed pollen in her pollen basket. There is no way to differentiate nectar from water foragers unless one collects the dancer and samples the fluid in her crop, which tends to disturb the dancing and other behaviours of the hive. However, as we are located in England, water foragers are relatively rare (<1%; see below for sucrose concentration data), even during the summer.

The four waggle runs were averaged to obtain a single duration and angle, which highly correlated to the duration and angle that would be obtained if one decoded and averaged all the waggle runs within a dance [30]. We converted duration into distance (meters) using a linear calibration model built for our honey bee population and landscape [33]. Using our own calibration curve instead of relying on the curve of von Frisch [20], as in previous studies, is important because the honey bee odometer is relative to the landscape over which they fly [34], [35] and may differ between bee strains [36].

Clocks radio-controlled for accuracy were also attached to each observation hive and visible in the video, which provided the exact time of each dance. Time of day was used in the calculation of solar azimuth using an Excel Macro (© W.F. Towne) Sun2007. We calculated angle+azimuth to obtain the final angle, which is measured as a clockwise heading from North. We then used the distance and heading to plot each dance (see below) each dance.

**Figure 1:**
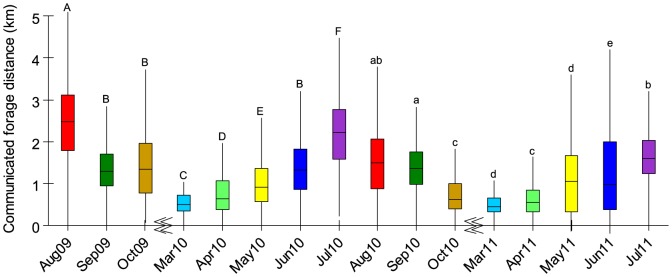
Monthly variation in honey bee foraging distance as determined from decoding 5097 waggle dances. Foraging distance varies significantly with month. The communicated distances were greater in summers (July & August) than springs (March & April) or autumns (September & October). Letters (capital = year 1 and lower case = year 2) display post-hoc results, where months that share letters do not significantly differ. Box lines report medians and lower and upper quartiles, and whiskers extend to either maximum and minimum data points or to 1.5 times the interquartile range. Breaks in the x axis indicate winter, when there is little or no foraging. Colours per month are consistent between figures, with the exception of Figure 4 heatmap.

In all, we decoded and analysed 2351 waggle dances from August 2009 to July 2010 (year 1) and 2746 from August 2010 to July 2011 (year 2; Figure 1). These dances were made for both nectar and pollen for most days in the bees' foraging season (March to October; 189 days of dance data from all 3 hives across the two years). Because there was not a consistent, significant difference between foraging distances for nectar and pollen (Couvillon, unpublished data), we did not differentiate between them for the purposes of this study. There were no unusual weather patterns (e.g., intense drought or flooding) during these two years. April 2011 was drier than usual, but not significantly so (Met Office Weather).

### Determining nectar sugar content

During 2012, we collected and chilled 10 returning foragers from two observation hives on days when the bees were actively foraging (113 days from March to October). The immobile bees had gentle pressure applied to their abdomens to cause them to regurgitate some of the nectar in their crop. Using a pipette, this was transferred to a handheld refractometer (Krüss, HR25-800) designed for small volumes to determine total sugar concentration (% w/w, °Brix). Readings of 0% indicate water collection, which we did not include in our analysis. Water was a rare occurrence (<5%), as English summers are not overwarm.

### Determining the effect of temperature on foraging distance

To determine if temperature affected foraging distance, we obtained from the National Met Office the daily maximum temperature for all study days (August 2009–August 2011) from Herstmonceux, which is the nearest weather station (approximately 27 km) and situated in a meteorologically similar location. For each week in which dances were decoded, we subtracted the lowest daily maximum temperature from the highest daily maximum temperature. For the same week, we subtracted the least far foraging distance, as communicated by the waggle dance, from the furthest foraging distance. If temperature alone drove the foraging distance pattern, we would expect these two differentials to correlate. However, there was no correlation, which indicates that there was no significant effect of temperature on foraging distance (Spearman Correlation, r = −0.125, p = 0.37).

### Plotting dances as probability distributions

Because of the presence of error in both components of the dance (duration and angle), it is impossible to predict exactforaging distances from decoded waggle runs [20], [30], [35]. Therefore, plotting dances as single points (distance+direction), as is generally done [26], [27], overestimates certainty about the true foraging location.

We used a Bayesian linear calibration model for distance vs. waggle run durations ([33]; scripts available in the reference's supplementary information online) using JAGS 3.3.0 [37] from within R [38] with the package rjags [39]. This allowed us to simulate distance distributions for the decoded waggle dances for unknown locations [33]. We then simulated the directional component using random von Mises samples (κ = 24.9) of equal size [33]. Lastly, we combined the calibrated distance distributions with the directional component (von Mises samples) to obtain a probability density for the foraging location communicated by the dance. In other words, a single dance can now be plotted not as a point, but as a colour-coded probability distribution. Then we took all the dances, simulated 1000 times as described above, and combined them per month. Combining many dances in such a way gives us an accurate visual of honey bee foraging patterns (see Figure 4 in [33]).

**Figure 2:**
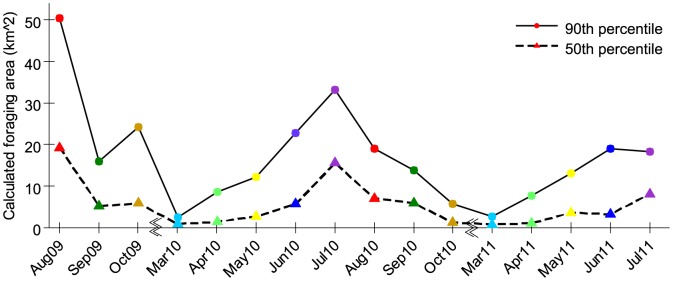
Calculated foraging area (km^2^) at 90^th^ and 50^th^ percentiles of distances indicated by waggle dances. Honey bees use an area approximately 22 times greater in the summer (August 2009 & July 2010) vs. spring (March 2010 & March 2011) and 6 times greater in summer vs. autumn (October 2009 & 2010). Breaks in the x-axis indicate winter, when there is little or no foraging. Colours per month are consistent between figures, with the exception of Figure 4 heatmap.

These simulated dance locations were binned across the landscape using the raster package in R, which determined the number of simulated dances per bin. We exported the resulting rasters as geo-referenced ESRI ASCII files from R into ArcGIS (version 10.0) with the package sp [40]. GIS automatically scaled and plotted per month from lowest (blue: 1 dance) to highest (red: 55–1292 dances) the number of dances per bin. We did not normalize across months because we wanted to show the locations of the relative hot spots in each month as indicated by the dancing bees. Foraging area greatly varied per month, and it is expected that dances per bin would also scale accordingly. Figure legends provide scale bars. Black concentric circles at 3 and 5 km were drawn around the geo-referenced laboratory location. White circles represent the 90^th^ and 50^th^ percentiles, as also shown in Figure 2. Aerial photographs were purchased from Getmapping PLC and imported as .jpeg and .jpw files into ArcGIS.

## Results

As shown in Figure 1, the mean foraging distances communicated by the dances vary significantly with month in both years. (Distances were square root transformed to obtain normality of residuals; one-way ANOVA for year 1, F_7,2343_ = 172.83, p<0.001; one-way ANOVA for year 2, F_7,2634_ = 112.24, p<0.001). This variation shows a general pattern with significantly greater distances in summers (defined as pre-autumnal/ivy bloom, average distance = 2156 m, August 09; July 2010; July 2011) than in early springs (average distance = 493 m, March 2010; March 2011) and autumns (defined as during autumnal/ivy bloom, average distance = 1275 m, Sep–Oct 2009; Sep–Oct 2010; Fig. 1). Summer is also the warmest season, but temperature was a non-significant predictor of distance (Materials and Methods, Spearman's Rank Order Correlation, r_s_ = −0.125, p = 0.37). Because we consider each dance to be our independent data unit, we did not include colony as a factor. However, we also analysed these data with a GLMM with colony as a random factor, and the results were the same.

These differences in foraging distance translate into very large differences in foraging area. We determined the 90^th^ and 50^th^ percentile foraging distances and estimated monthly foraging area as a circle with this radius (Fig. 2). The calculated foraging area used by the bees in the summer (August 2009) was 22 and 26 times greater than early spring (March 2010) at the 90^th^ and 50^th^ percentiles, respectively. In July 2010, the calculated foraging area was 14 and 26 times greater than early spring (March 2011) at the same percentiles. Together, this gives a 22-fold average ratio in foraging area for summer vs. early spring over the two years. The calculated foraging area used by colonies in summer (August 2009) was also 2 and 3 times greater than autumn (October 2009) at the 90^th^ and 50^th^ percentiles, respectively. In July 2010, the calculated foraging area was 6 and 14 times greater than autumn (October 2010) at the same percentiles. Together, this gives a 6-fold average ratio in foraging area for summer vs. autumn over the two years.

**Figure 3:**
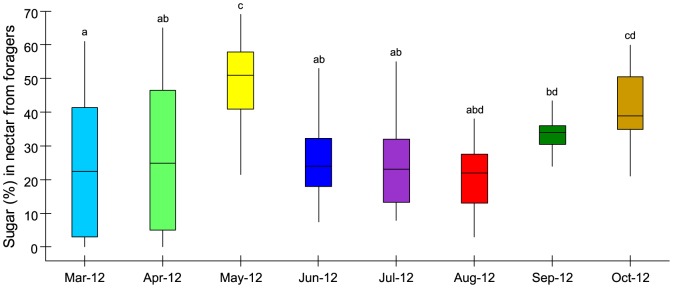
Sugar content of nectar brought back to the hives by returning foragers. Median sugar content is highest in May, September, and October and is lowest in March, April, and June to August. The third quartile is higher in spring than summer. Letters display post-hoc results, where months that share letters do not significantly differ. Statistics were done on transformed data, but the Figure displays the untransformed data. Box lines report medians and lower and upper quartiles, and whiskers extend to either maximum and minimum data points or to 1.5 times the interquartile range. Breaks in the x axis indicate winter, when there is little or no foraging. Colours per month are consistent between figures, with the exception of Figure 4 heatmap.

Our data also show that summer is also a season when nectar sugar content is not significantly higher. Sugar content (%) is a correlative measure of nectar quality, as sweeter nectar contains more energy, and bees have evolved great sensitivity to this metric [23], [24], [41]. We found that sugar concentration in nectar varies significantly with month (% sugar, as response, was arcsine-transformed to obtain normality of residuals; One-way ANOVA, F_7,282_ = 13.93, p<0.001; Fig. 3). In June, July and August, the median and range of sugar content is low. The median sugar content is also low in March and April. However, spring sugar concentration range is wide, showing that better quality nectar is also available (and at closer distances) to foragers. Taken together, the data show that in summer compared to spring or autumn, the bees fly further to bring back nectar that is not better in quality.

**Figure 4:**
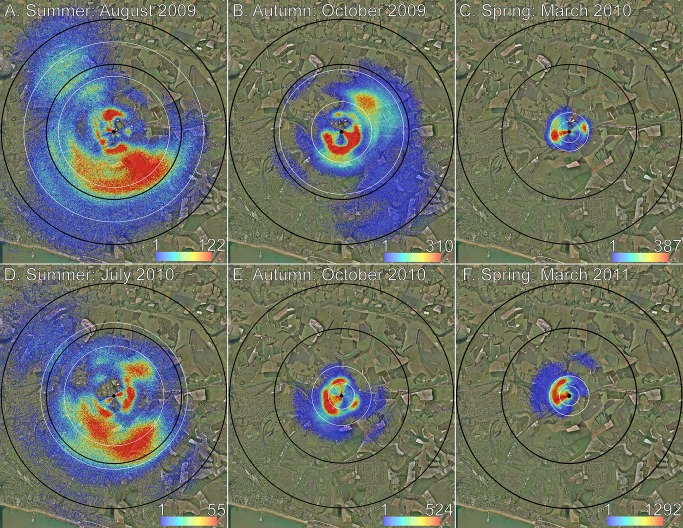
Distribution and density of foraging locations as determined by waggle dances. Each dance is simulated 1000 times to incorporate the error inherent in dance information. Colour denotes how many dances fall within 25×25 m bins. Black circles are 3 and 5 km from the hive locations (centre black dot). White circles indicate the areas corresponding to the 90^th^ and 50^th^ foraging distance percentiles. Foraging range, containing a diversity of urban and rural land-types, extends the furthest (**A, D**) during summer (August 2009, n = 439×1000 dances; July 2010, n = 340×1000 dances), less far (**B, E**) in autumn (October 2009, n = 401×1000 dances; October 2010, n = 231×1000 dances), and least far (**C, F**) in early spring (March 2010, n = 114×1000 dances; March 2011, n = 195×1000 dances) when flowers are readily available.

The need for bees to use the landscape more widely in summer is especially striking when the distance and direction components of the dance vectors are mapped. Figure 4 shows where the honey bees foraged in two summer months, two autumn months, and two spring months. Each dance is plotted to include the error inherent in the dance (see Materials and Methods), resulting in a novel visualization method that maps the joint probability distributions of all foraging from the three study colonies [33].

## Discussion

Here we have shown that honey bees, foraging over a landscape that is typical of most of the Western world, must travel further, covering a significantly larger area, in the summers (2156 m, 15.2 km^2^) compared to springs (493 m, 0.8 km^2^) or even autumns (1275 m, 5.1 km^2^) to collect forage that is not of better quality. Our study is necessarily set in one location to investigate the spatio-temporal changes in foraging patterns; however, these data also demonstrate that dancing bees may act as indicators, pinpointing in particular what months are representing relative dearth in forage availability.

Because not all foragers make waggle dances, dance decoding does not give information about all the foraging sites currently being used by a honey bee colony; rather, waggle dances are filtered information that communicate the most profitable feeding locations known to a colony at that time [42]. Foraging honey bees are very sensitive to relative energetic reward [23], [24], which heavily weighs flight cost. Patches of better quality, because they are closer or possess higher quality nectar, will be valued higher by honey bees and generate more dancing and more repeated waggle runs within each dance [24], [41], all of which cause increased recruitment. Each dance represents economically savvy advice for a colony's unemployed foragers as to where to collect food. Dance decoding, therefore, provides an integrated picture of the best feeding locations. The fact that waggle dances in August and July advertise patches at the greatest distances indicates that summer is the most challenging season to find food in the study landscape.

In contrast to summer, during spring the bees danced for much closer locations, mostly within 500 m from the hives (Fig. 4C, 4F). In many temperate habitats, spring is a season of great flower abundance, with woodland flowering species that bloom before the tree canopy matures, including trees, shrubs, perennial herbs and annuals [43]. Abundant flowers mean that bees are able to forage and to recruit locally. Additionally, even though the weather in autumn is less favourable than in summer, the dance decoding indicates that foraging conditions actually improve from summer to autumn. This is due to ivy (*Hedera* spp.), a common European flowering plant that is very abundant in both urban and rural settings. In the study area, ivy begins to bloom in August, with the first flowers seen on 29 August, 2009 and 14 August, 2010, and peaks in September and October. Honey bees feed almost exclusively on ivy for both nectar and pollen in the autumn [44], and its ubiquity means that they can forage closer than in summer (Fig. 4B, 4E). Ivy nectar is also high in sugar, c. 45%, which most likely accounts for the improved quality of autumn nectar compared to summer [44].

What general lessons can be learned from our study, which was necessarily set in a particular location? Historically, the landscape contained more habitats, such as hay meadows with abundant summer-flowering plants. Since World War II, these have been much reduced due to agricultural intensification [9], [13]–[16], [45]. Concurrent with these reductions, the number of managed honey bee hives has decreased 75% in Great Britain in the past century [5], which mirrors the drop in other flower-visiting insects, including bumble bees, solitary bees, butterflies, and hoverflies [1]–[4]. These declines and their link to landscape changes have generated much attention, including initiatives by governments and commercial organizations, such as seed companies, to increase forage [46]. However, the information on how to help bees appears not always to be soundly based on scientific data, such as recommendations to grow winter-blooming garden plants, when most bee species (including honey bees) are dormant, or the description, without supportive data, of a June “hungry gap” [47]. The UK Royal Horticultural Society, in its “Perfect for Pollinators” campaign, recently advised the planting of garden flowers to bloom during “the entire period of bee activity”. While it is certainly correct that bees require flowers throughout the entire foraging season, the herculean task of increasing the availability of forage would be more manageable and cost-effective if aid could be better targeted. Our study suggests that in a particular location, the greatest challenge for finding food will be concentrated in a portion of the much longer foraging season. The question then becomes how do we identify these periods of relative food dearth? Beekeepers sometimes point to changes in hive weight as identifying periods of forage dearth and abundance; however, this practice is confounded by hive size and by weather. A second way in which to obtain directly the data on the amount of forage available in a landscape-wide area would be, with great effort, to count competing flower-visiting insects and flowers and determine nectar and pollen availability. In contrast, the alternative, as we have done in this study, is to use honey bee dances to obtain a picture that already integrates all these factors.

Our study region is temperate and similar to most of Great Britain and parts of Europe and North America possessing strong spring flowering, some autumn flowering (e.g., ivy in Europe; golden rod, asters in the USA), and a mixed landscape of urban and rural habitats with large agricultural areas of monocrops. Therefore, our particular results of summer foraging challenges could be generally applicable. However, of more widespread practical importance is our general result: we show that honey bees can act as indicators, and dance decoding can be implemented to survey landscapes to determine when forage is hardest to locate. Such information will help place recommendations to help bees and flower-visiting insects onto a more solid foundation based on empirical evidence.

Determining where foraging animals collect food is valuable in conservation work, and recent years have witnessed an explosion in the use of GPS trackers for this purpose [48]. Although insects are too small for these technologies, trackers are actually unnecessary with the honey bee, which is the only animal that directly tells eavesdropping researchers where it has collected food. Additionally, although the honey bee is only one of many flower-visiting insects, it is a generalist forager, and flower-rich locations visited by honey bees will be visited by other flower-visiting insects as well [44], [49]. This makes the evidence for seasonal forage scarcity widely relevant for insect pollinators, especially as there is a valuable pollination synergy between honey bees and other bees [50]. The honey bee is the only animal who tells you where it has collected food. Here we have shown that listening to the bees will allow us to better direct efforts to make our landscape more insect-friendly.
